# Frequent high-level rifaximin resistance in *Escherichia coli* associated with long-term treatment of patients with liver cirrhosis: a prospective, controlled study

**DOI:** 10.1128/spectrum.03347-24

**Published:** 2025-10-13

**Authors:** L. Boll, W. V. Kern, S. Schuster, M. Schultheiß, C. Schneider, M. Vavra, R. Thimme, S. Rieg, J. Camp

**Affiliations:** 1 Division of Infectious Diseases, Department of Medicine II, Medical Centre – University of Freiburg, Faculty of Medicine, University of Freiburg 9174, Freiburg, Germany; 2 Department of Medicine II, Medical Centre – University of Freiburg, Faculty of Medicine, University of Freiburg 9174, Freiburg, Germany; 3 Berta-Ottenstein Programme, Faculty of Medicine, University of Freiburg 9174, Freiburg, Germany; Ascension St. John Hospital, Detroit, Michigan, USA

**Keywords:** antimicrobial activity, liver cirrhosis, rifaximin

## Abstract

We conducted a prospective, controlled study to assess the prevalence of high-level rifaximin resistance in intestinal *Escherichia coli (E. coli*) among patients with liver cirrhosis (LC). Rectal swabs were collected from consenting adult LC patients at Freiburg University Medical Center. Swabs were cultured on MacConkey agar plates supplemented with rifaximin at two concentrations (32 µg/mL and 256 µg/mL), and pink/red colonies were identified as *E. coli* using standard methods. Minimum inhibitory concentrations (MICs) of rifaximin and a panel of additional agents were determined using microdilution assays. Of the 100 patients enrolled, 49 received rifaximin (median duration 22 weeks), and 51 served as controls. Patients receiving rifaximin had more advanced LC (Child B/C, 75% vs. 50%) and more frequent concurrent antibiotic use (22% vs. 12%). High-level rifaximin-resistant *E. coli* strains were recovered from 67% (33/49) of rifaximin-treated patients, compared to 12% (6/51) of controls (*P* < 0.001). Cross-resistance to rifampicin was complete, with no clear associations with other resistance phenotypes. A subset of five high-level resistance swabs was assessed for homogeneity of colonization by randomly picking *E. coli* strains from a non-supplemented MacConkey agar plate. Homogeneous colonization with high-level rifaximin-resistant *E. coli* was found in 80% of cases. The prevalence of high-level rifaximin-resistant intestinal *E. coli* in LC patients on long-term rifaximin treatment is extremely high (67% vs. 12%). While rifaximin may remain effective in preventing hepatic encephalopathy (HE), its potential use in preventing spontaneous bacterial peritonitis might be severely limited by the emergence of resistance.

**IMPORTANCE:**

This study highlights the emergence of antibiotic-resistant *E. coli* as an important consequence of long-term rifaximin use in patients with liver cirrhosis: the development of antibiotic resistance in gut bacteria. Rifaximin is commonly used to prevent complications like hepatic encephalopathy (HE) by targeting gut bacteria. However, this research shows that prolonged use can lead to the emergence of highly resistant *E. coli* strains, potentially compromising the drug’s effectiveness and limiting its utility in preventing HE and infections, such as spontaneous bacterial peritonitis. By identifying specific resistance patterns and genetic mutations, this study highlights the need for cautious use of rifaximin and further research into balancing its benefits with the risks of resistance. These findings contribute to a broader understanding of how antibiotics affect gut bacteria over time and offer a basis for exploring strategies to preserve the effectiveness of this important medication for patients with advanced liver disease.

## INTRODUCTION

Rifaximin is a non-absorbable rifamycin developed for the treatment of traveler’s diarrhea now commonly used in the management of patients with liver cirrhosis (LC), irritable bowel syndrome, or Crohn’s disease (1–3). Due to its minimal systemic absorption and high achievable concentration in the colon, rifaximin specifically targets the gut microbiota, reducing bacterial overgrowth and the production of toxins such as ammonia (1, 3). Its role in preventing hepatic encephalopathy (HE) has been well-established (4), and several studies have yielded promising results regarding the use of rifaximin in the prevention of spontaneous bacterial peritonitis (SBP) (5, 6).

The potential for the development of antimicrobial resistance associated with rifaximin use has been the subject of considerable debate (7–9). Rifaximin-resistant strains of *E. coli* and other gut bacteria have been reported, raising questions about the long-term implications of rifaximin therapy on resistance patterns within the gut microbiota (10, 11). Resistance to rifaximin could potentially undermine its effectiveness in preventing both HE and SBP, particularly in patients with advanced liver disease, where the risk of infection is already heightened.

The present study investigates the prevalence and characteristics of rifaximin-resistant *E. coli* strains in LC patients undergoing long-term rifaximin treatment. By comparing these patients with a control group of patients with LC who did not receive rifaximin, we aimed to elucidate the impact of rifaximin on bacterial resistance patterns.

## MATERIALS AND METHODS

The research project was a monocentric cross-sectional study conducted at Freiburg University Medical Center. The study was approved by the ethics committee of the University of Freiburg Medical Center under protocol number 21-1193 on December 14, 2021. Included patients were required to be at least 18 years, have LC, and provide informed consent to participate in the study. The case group included patients with any Child-Pugh stage who had received rifaximin prophylaxis for at least four weeks. The control group consisted of patients with any Child-Pugh stage without rifaximin prophylaxis. All patients provided written informed consent. Baseline epidemiology and prescription of additional antibiotics in the last four weeks were recorded.

We collected a rectal swab from the patients using a liquid elution swab system containing Amies broth and simultaneously inoculated 50 µL of the swab fluid onto each of three agar plates: (1) a regular MacConkey agar plate (selective for coliforms and other intestinal bacteria due to the presence of bile salts); (2) a MacConkey agar plate supplemented with rifaximin at a concentration of 32 µg/mL; and (3) a MacConkey agar plate with 256 µg/mL of rifaximin. The regular MacConkey agar plate served as growth control. As a positive control, we used the rifaximin-resistant *E. coli* strain 3-AG100 in parallel (12). The bacterial growth on the plates after 24 hours of incubation was evaluated on a scale from 0 (no growth) to 3 (abundant growth). Patients whose swabs did not show any growth on the native MacConkey agar were excluded. Only bacteria growing on the 256 µg/mL rifaximin plate were considered to exhibit high-level resistance to rifaximin in the agar screening. From each patient, three red or pink colonies indicating *E. coli* (due to its ability to catabolize lactose resulting in pH change) were selected for further investigation. If the isolation of single colonies was not possible, dilutions of the original swab were prepared and plated. The species confirmation was performed by MALDI-TOF and additionally through analysis of sequencing data with the KmerFinder 3.2 service (https://cge.food.dtu.dk/services/KmerFinder/) (13, 14). The selected and confirmed *E. coli* colonies were examined for their antibiotic resistances using VITEK. In addition, the MICs of various antibiotics were determined by microdilution assays in cation-adjusted Mueller-Hinton broth (MH II), with and without the efflux pump inhibitors Phe-Arg β-naphthylamide (PAβN) and 1-(1-naphthylmethyl)-piperazine (NMP), at concentrations of 25 µg/mL and 100 µg/mL, respectively. The rifaximin MICs obtained through this method served as a confirmation test for the results of the agar screening. The MIC assays were carried out at least in duplicate and according to EUCAST standard procedures.

For next-generation sequencing (NGS), DNA isolation from bacteria, library preparation (Illumina DNA tagmentation), and sequencing were carried out by Microsynth (Balgach, Switzerland) on an Illumina NovaSeq, SP, 2 × 150 bp (target: 1 Gb per sample). Mutations were detected by mapping the trimmed reads to the NCBI complete genome RefSeq NC_000913.3 of *Escherichia coli* str. K-12 substr. MG1655 (https://www.ncbi.nlm.nih.gov/nuccore/556503834), using the CLC Genomics Workbench Version 11.0.1 (QIAGEN Aarhus, Denmark). Sequence types and the presence of acquired resistance genes were determined using the MLST 2.0 (https://cge.food.dtu.dk/services/MLST/) (15–17) and the ResFinder 4.6.0 services (http://genepi.food.dtu.dk/resfinder) (18, 19).

The swab system (eSwab™) was from COPAN ITALIA S.P.A. (Brescia, Italy); MacConkey broth was from Carl Roth (Karlsruhe, Germany); MH II broth from Becton Dickinson (Franklin Lakes, USA); 1-(1-naphthylmethyl)-piperazine from CHESS (Mannheim, Germany); Phe-Arg β-naphthylamide, rifaximin, and rifampicin from Merck (Darmstadt, Germany); and the precast 96-well MIC assay plates pre-filled with the dehydrated antibiotics—levofloxacin, moxifloxacin, tetracycline, minocycline, tigecycline, cefuroxime, linezolid, chloramphenicol, clindamycin, novobiocin, rifampicin, rifaximin, gentamicin, and azithromycin (MICRONAUT-S plates from Merlin Diagnostika, Bornheim-Hersel, Germany).

Statistical tests were used as appropriate (Student’s *t*-test, Fisher’s exact test). All calculations were performed with R 4.3.1 and RStudio Server 2023.06.1.

## RESULTS

During the study period, 100 patients with LC were included. Among these, 49 received rifaximin (550 mg bid, one patient sid, two 200 mg bid) as part of their treatment plan (RFX group) and 51 served as controls. Patients in both groups were of comparable age and were predominantly male (Table 1). The severity of liver disease, as assessed by Child-Pugh classification, differed significantly between groups (*P* = 0.025, Fisher–Freeman–Halton exact test). A higher proportion of control patients had Child-Pugh class A liver cirrhosis (49% vs. 24%), while Child-Pugh class C cirrhosis was more frequent in the RFX group (20% vs. 8%). Rates of TIPS placement, however, differed only marginally (57.1% RFX vs. 50.9% control, *P* = 0.554). By the end of the observation period, 22% of RFX patients had died compared to 8% in the control group (*P* = 0.050, Fisher’s exact test). Median time of RFX treatment was 22 weeks.

**TABLE 1:** Patient characteristics^*a*^

	Cases (*n* = 49)	Controls (*n* = 51)	*P*-value
Median age in years (range)	61 (38–79)	60 (38–81)	0.920
Male gender, *n* (%)	30 (61%)	33 (65%)	0.836
TIPS present, *n* (%) 28	28 (57%)	26 (51%)	0.554
LC severity, *n* (%)			0.022
Child-Pugh A	12 (24%)	25 (49%)	
Child-Pugh B	27 (55%)	22 (43%)	
Child-Pugh C	10 (20%)	4 (8%)	
Inpatient (vs. outpatient)	30 (61%)	19 (37%)	0.027
Median duration of Rfx treatment, weeks (range)	22 (4–320)	–^*b*^	–
Additional antibiotics at the time of sampling, *n* (%)	11 (22%)	6 (12%)	0.189

^*a*^ Rfx, rifaximin; TIPS, transjugular intrahepatic portosystemic shunt; LC, liver cirrhosis. For continuous variables, Mann–Whitney *U *test was used. For categorical variables, Fisher’s exact test was applied, except for LC severity, where the Fisher–Freeman–Halton exact test was used.

^*b*^ “–” indicates absence of mutation.

One hundred percent of swabs (49/49) obtained from the RFX group exhibited bacterial growth at the lower concentration of 32 µg/mL rifaximin. Confirmed *E. coli* strains exhibiting high-level resistance in the agar screening (i.e., growing on the 256 µg/mL rifaximin plate) were recovered in 33/49 (67 %) cases compared to 6/51 (12%, *P* < 0.001) in the control group (Table 2). All strains identified as highly resistant by agar screening were subsequently confirmed by microdilution to have rifaximin MICs of greater than 256 µg/mL, except for one strain from the control group which showed an MIC of 16 µg/mL and was therefore not considered highly resistant. Rifampicin MICs exceeded 256 µg/mL in all highly resistant *E. coli* strains (33 case strains, six control strains), indicating complete cross-resistance with rifampicin.

**TABLE 2:** Agar screen on MacConkey agar plate with rifaximin (Rfx) (for categorical variables, Fisher’s exact test was applied)

	Cases (*n* = 49)	Controls (*n* = 51)	*P*-value
Bacterial growth at, *n* (%)			
32 µg/mL Rfx	49 (100 %)	26 (51 %)	<0.001
256 µg/mL Rfx	45 (92 %)	7 (14 %)	<0.001
Confirmed *E. coli* on, *n* (%)			
32 µg/mL Rfx plates	34 (69 %)	14 (27 %)	<0.001
256 µg/mL Rfx plates	33 (67 %)	6 (12 %)	<0.001

Additionally, we examined whether longer treatment duration was associated with the recovery of rifaximin-resistant *E. coli*, but found no significant difference in median duration between patients with and without resistant isolates (Wilcoxon rank-sum test, *P* > 0.050). Stratification by treatment duration (≤1 year vs. >1 year) showed a non-significant trend toward higher resistance with longer intake (OR 1.86, 95% CI: 0.38–12.41; *P* = 0.500, Fisher’s exact test).

Among the 33 rifaximin-resistant *E. coli* isolates from the case group, a variety of mutations in the *rpoB* gene, which confer resistance to rifamycins, were identified. The most common mutation was D516N, present in four isolates, while combinations of D516N with other mutations, such as Q148L and R1211T, and the acquired resistance genes ARR-2 and ARR-3 were observed in an additional five isolates. The S574F mutation appeared in three isolates, with one isolate harboring both S574F and Q148L (Table 3). In the control group, mutations associated with high-level rifaximin resistance were much less common. Two isolates exhibited the D516N mutation, while single mutations, such as D516Y, Q148L, and S509R, were identified in separate isolates. Additionally, two control isolates carried the ARR-2 and ARR-3 resistance elements without accompanying *rpoB* mutations.

**TABLE 3:** Mutations associated with high-level resistance to rifaximin in *E. coli* clinical isolates with distinct multilocus sequence typing *in silico* serotypes

	Case isolates	Control isolates
D516N	4	2
D516*N* + Q148L	1	–
D516*N* + R1211T	1	–
D516*N* + ARR-2	1	–
D516*N* + ARR-3	1	–
D516Y	3	1
S574F	3	–
S574F + Q148L	1	–
Q513L	2	–
P564L	2	–
H526Y	2	–
H526Q	1	–
Q148L	1	1
S509R	–	1
ARR-2 alone	1	–
ARR-3 alone	1	–

In order to further characterize the recovered strains, we also obtained MICs of other antimicrobial substances. We found that strains from the RFX group tended to harbor more and more diverse antimicrobial resistance patterns than the strains from the control group (Table 4). Resistance against levofloxacin was detected in 4/33 (12 %) in the RFX group compared to 0/6 (0 %) in the control group, and resistance against second-generation cephalosporins was also more common in the RFX group (18% vs. 0%). Fluoroquinolone and beta-lactam MICs in the presence of known efflux pump inhibitors (EPI) showed no significant MIC reductions, whereas the MICs of clindamycin, minocycline, and linezolid were significantly reduced (Table 5).

**TABLE 4:** *In vitro* Co-Resistance in high-level rifaximin-resistant *E. coli* (as identified by agar screening and confirmed by MIC testing)^*a*^

	Case isolates (*n* = 33)	Control isolates (*n* = 6)
Microdilution results, *n* (%)		
Levofloxacin >1 µg/mL	4; 12%	0
Tetracyclin >2 µg/mL	20; 61%	3; 50%
Tigecyclin >0.5 µg/mL	0	0
Cefuroxim >8 µg/mL	6; 18%	0
Gentamicin >2 µg/mL	4; 12%	1; 17%
Azithromycin >16 µg/mL	16; 47%	3; 50%
Vitek two system results, %		
Ampicillin >8 µg/mL	7; 21%	2; 33%
Piperac./Tazob. >8 µg/mL	0	0
Cefotaxim >2 µg/mL	3; 9%	0
Cotrimoxazol >4 µg/mL	2; 6%	0

^*a*^ Breakpoints are specified according to EUCAST criteria. Where breakpoints were not available, breakpoints were inferred from related organisms: tetracycline breakpoints were based on *Haemophilus* and *Moraxella* spp., and azithromycin breakpoints were derived from *Salmonella* spp.

**TABLE 5:** Efflux phenotype in high-level rifaximin-resistant *E. coli^a^*

	Case isolates		Control isolates	
% of ≥4-fold reduced MIC by:	NMP	PAβN	NMP	PAβN
Chloramphenicol	38	44	50	57
Linezolid	100	100	100	100
Moxifloxacin	3	9	7	21
Clindamycin	94	100	100	100
Minocycline	85	88	64	93
Novobiocin	12	85	0	100
Azithromycin	56	68	57	43

^*a*^ MIC, minimum inhibitory concentration; NMP, 1-(1-naphthylmethyl)-piperazine; PAβ*N*, PheArg β-naphthylamide.

To further assess the homogeneity of colonization with high-level resistant *E. coli*, we randomly isolated additional strains from a subset of patients in the case group (*n* = 5) and the control group (*n* = 4) from a non-supplemented MacConkey agar plate. MIC testing revealed a homogeneous colonization with high-level resistant *E. coli* in 80% of the analyzed patients in the case group. In the control group, 50% of the *E. coli* from the supplemented MacConkey agar plate exhibited high-level resistance.

## DISCUSSION

Drawing on prospectively evaluated data, this study demonstrates that the prevalence of high-level rifaximin-resistant *E. coli* in LC patients on long-term rifaximin treatment is extremely high (67% vs. 12%, *P* < 0.001). Despite its growing importance in the management of patients with LC (20), antimicrobial resistance of coliforms against rifaximin has not been studied in this collective. While data from irritable bowel disease patients showed no increased resistance levels of gut microbes after repeat short-term courses of rifaximin (10, 11), our results are more in line with early data gathered from a small sample of healthy volunteers which showed resistance levels of a variety of bacterial strains in the range of 30–90% after exposure to short-term treatment with rifaximin (21). Additionally, these studies analyzed only short-term courses of rifaximin treatment, whereas in our cohort, the median duration of treatment at study inclusion was 22 weeks. Kothary also found that prior exposure to rifaximin increased the likelihood of the presence of rifaximin-resistant *E. coli* in IBS patients, albeit their reported rate (25%) was considerably lower than in our study (7). Our findings are further corroborated by *in vitro* studies which show that resistance against rifaximin can develop after a single exposition step (7, 22). The discrepancies in reported resistance rates may also be partly attributable to the differences in underlying diseases, as LC patients, particularly in advanced stages of the disease, often have altered gut microbiota, impaired immune responses, and high rates of hospitalization, rendering them prone to infections with multidrug-resistant organisms (4, 23, 24).

The clinical implications of increased rifaximin resistance depend on the context of use, especially in populations such as LC patients, where the drug is routinely used to prevent hepatic encephalopathy (HE). The high prevalence of rifaximin-resistant *E. coli* observed in our cohort—coupled with the homogeneous colonization—may potentially limit its future utility in LC management. While rifaximin is not commonly used for direct infection control, its efficacy in reducing HE is attributed to its impact on gut microbiota, specifically reducing ammonia-producing bacteria (25). However, our data indicate that the emergence of high-level resistance may lead to less effective microbiota modulation, potentially diminishing rifaximin’s protective effect against HE.

Although not commonly used in this indication, several studies have shown the efficacy of rifaximin in SBP prevention to be comparable to that of fluoroquinolones (5, 6, 26, 27). Due to the rapid development of resistance against fluoroquinolones, therapeutical alternatives are much needed. While there is some discussion on whether antimicrobial activity is the primary mechanism of rifaximin in HE prevention (8), the prevention of SBP indeed relies on reduction of viable microbes, especially *E. coli*. Causative agents of SBP in patients under fluoroquinolone prophylaxis are likely to be resistant (28). Our data show that the development of rifaximin resistance is very common among patients under prophylaxis, and hence, a similar loss of protection against SBP over time seems plausible.

The detected mutations in the *rpoB* gene, particularly D516N and S574F, as well as the accessory genes *arr2/3* are well known to confer high-level rifaximin resistance in *E. coli* isolates and have been documented previously (7, 29–31). The predominance of the D516N mutation, often in combination with other mutations like Q148L or resistance elements ARR-2 and ARR-3, suggests a multifactorial resistance pattern emerging under rifaximin exposure. These mutations affect the rifamycin-binding site on the RpoB subunit of RNA polymerase, reducing drug efficacy (29). The more complex mutation patterns observed in the case group may indicate that prolonged rifaximin could drive the selection of more diverse and potent resistance mechanisms.

Furthermore, our study’s findings regarding the prevalence of resistance against levofloxacin and cephalosporins in the RFX group suggest that rifaximin exposure may have broader implications for antimicrobial resistance development beyond the rifamycin class. These trends are consistent with the literature documenting the collateral effects of non-systemic antibiotics on gut microbiota (32, 33). Our data also show that the high-level rifaximin-resistant *E. coli* strains exhibit resistance profiles that are partially amenable to EPI treatment. Efflux pumps are known to contribute significantly to multidrug resistance by actively expelling antibiotics from bacterial cells, reducing intracellular drug concentrations, and consequently diminishing the efficacy of antibiotics (34, 35). While other resistance mechanisms, such as mutations in target sites or enzymatic degradation, are likely responsible for resistance against fluoroquinolones and beta-lactams in this cohort, the significant reduction in MICs of clindamycin, minocycline, and linezolid in the presence of EPIs provides evidence that efflux pumps contribute to resistance against these antibiotics. This highlights the possibility of efflux pump overexpression as a secondary mechanism contributing to the multidrug-resistant profiles observed in the RFX group. Efflux pump-mediated resistance can result in cross-resistance to a variety of structurally unrelated antibiotics, further complicating treatment options (36). In this case, the identification of efflux-related resistance suggests that rifaximin exposure could indirectly select for strains with broader multidrug-resistant profiles, as these pumps can be co-regulated with other resistance genes under selective pressure from antibiotics (35).

Our study has some limitations. Because rifaximin prophylaxis is commonly used in more advanced stages of liver disease, patients in the case group tended to be more severely affected, which might have entailed a higher rate of baseline antimicrobial resistance. Furthermore, we were unable to observe a case of spontaneous bacterial peritonitis in our cohort during the follow-up period, likely due to the relatively small sample size.

To conclude, our findings suggest that the development of rifaximin resistance in *E. coli* under prophylaxis is a common occurrence. Further research is needed to ascertain the clinical impact of this finding. In particular, cycling or dose adjustment strategies could minimize resistance emergence while maintaining therapeutic efficacy. Further studies are also required to assess the potential transmission of resistant strains between patients and whether environmental or infection control measures are necessary to prevent wider dissemination of these resistant organisms in healthcare settings.
